# Molecular evolution of urea amidolyase and urea carboxylase in fungi

**DOI:** 10.1186/1471-2148-11-80

**Published:** 2011-03-29

**Authors:** Pooja K Strope, Kenneth W Nickerson, Steven D Harris, Etsuko N Moriyama

**Affiliations:** 1 School of Biological Sciences, University of Nebraska, Lincoln, NE 68588, USA; 2 Department of Plant Pathology, University of Nebraska, Lincoln, NE 68588, USA; 3 Center for Plant Science Innovation, University of Nebraska, Lincoln, NE 68588, USA

## Abstract

**Background:**

Urea amidolyase breaks down urea into ammonia and carbon dioxide in a two-step process, while another enzyme, urease, does this in a one step-process. Urea amidolyase has been found only in some fungal species among eukaryotes. It contains two major domains: the amidase and urea carboxylase domains. A shorter form of urea amidolyase is known as urea carboxylase and has no amidase domain. Eukaryotic urea carboxylase has been found only in several fungal species and green algae. In order to elucidate the evolutionary origin of urea amidolyase and urea carboxylase, we studied the distribution of urea amidolyase, urea carboxylase, as well as other proteins including urease, across kingdoms.

**Results:**

Among the 64 fungal species we examined, only those in two Ascomycota classes (Sordariomycetes and Saccharomycetes) had the urea amidolyase sequences. Urea carboxylase was found in many but not all of the species in the phylum Basidiomycota and in the subphylum Pezizomycotina (phylum Ascomycota). It was completely absent from the class Saccharomycetes (phylum Ascomycota; subphylum Saccharomycotina). Four Sordariomycetes species we examined had both the urea carboxylase and the urea amidolyase sequences. Phylogenetic analysis showed that these two enzymes appeared to have gone through independent evolution since their bacterial origin. The amidase domain and the urea carboxylase domain sequences from fungal urea amidolyases clustered strongly together with the amidase and urea carboxylase sequences, respectively, from a small number of beta- and gammaproteobacteria. On the other hand, fungal urea carboxylase proteins clustered together with another copy of urea carboxylases distributed broadly among bacteria. The urease proteins were found in all the fungal species examined except for those of the subphylum Saccharomycotina.

**Conclusions:**

We conclude that the urea amidolyase genes currently found only in fungi are the results of a horizontal gene transfer event from beta-, gamma-, or related species of proteobacteria. The event took place before the divergence of the subphyla Pezizomycotina and Saccharomycotina but after the divergence of the subphylum Taphrinomycotina. Urea carboxylase genes currently found in fungi and other limited organisms were also likely derived from another ancestral gene in bacteria. Our study presented another important example showing plastic and opportunistic genome evolution in bacteria and fungi and their evolutionary interplay.

## Background

Fungi exhibit great metabolic flexibility in the diversity of carbon and nitrogen sources they can use. We have been especially interested in their nitrogen sources, most recently urea [1,2]. In a previous study [1], a dichotomy was observed with regard to urea utilization in fungi. Hemiascomycetes (yeasts and yeast-like fungi; the majority belongs to the class Saccharomycetes of the phylum Ascomycota) possess the urea amidolyase (*DUR1,2*; Degradation of URea) genes whereas all other fungi examined possess the nickel-containing urease sequences. Urea amidolyase is an energy dependent biotin-containing enzyme. It is encoded by the *DUR1,2 *gene and was first characterized in the yeast *Candida utilis*, now known as *Pichia jadinii *[3]. The activity of this enzyme was also detected in green algae such as *Asterococcus superbus *and *Chlamydomonas reinhardii*. Urease and urea amidolyase activities were not observed together in the same green algal species; it was either one or the other [4,5]. This cytoplasmic, biotin-dependent enzyme [6] consists of a single polypeptide chain with regions for urea carboxylase (EC 6.3.4.6) and allophanate hydrolase (also known as amidase; EC 3.5.1.54) activity. Two adjacent genes (*DUR1 *and *DUR2*) were originally considered to encode the two enzymes; but later they were renamed as a single gene, *DUR1,2 *[7].

Urea amidolyase breaks down urea into ammonia and carbon dioxide in a two-step process, while urease (EC 3.5.1.5) does this in a one-step process [1] as shown in the following equations:(1)(2)(3)

**Figure 1:**
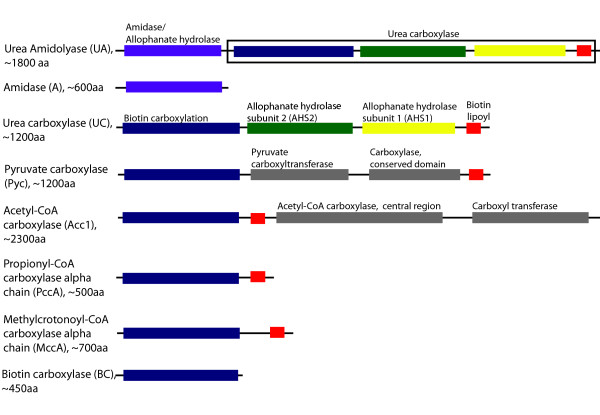
**Domain structures of urea amidolyase and related proteins**. Proteins that share the amidase (allophanate hydrolase) or the biotin-carboxylation domain are listed. The domains colored in grey are those that are not shared among these proteins. The domain structures are based on the InterPro protein domain database [38]. The abbreviations and approximate amino-acid lengths are given with the protein names. Amidase and urea carboxylase sequences exist as domains within the urea amidolyase protein or as single proteins by themselves. Similarly, the biotin-carboxylation sequence exists as a domain in various proteins as well as by itself as in the biotin-carboxylase protein.

There are two forms of urea amidolyase proteins. Figure 1 shows the domain structure of urea amidolyase and related proteins. A shorter form of urea amidolyase is known as urea carboxylase, and has no amidase domain attached to it. This protein is found in several fungal species [1], green algae [8], and has been also characterized in bacteria [9].

The urea carboxylase protein (as well as the domain) is further divided into sub-domains: the biotin-carboxylation domain, allophanate hydrolase subunit 1 (AHS1) domain, allophanate hydrolase subunit 2 (AHS2) domain, and the biotin-lipoyl domain (Figure 1). The function of the AHS1 and AHS2 domains is still unknown. The biotin-carboxylation domain and the biotin-lipoyl domain of urea carboxylase are commonly found in various other carboxylases including pyruvate carboxylase (Pyc), methylcrotonoyl-CoA carboxylase (MccA), acetyl-CoA carboxylase (Acc1), and propionyl-CoA carboxylase (PccA) [10].

In Navarathna *et al. *[1], we suggested that urea amidolyase likely arose before the divergence of the hemiascomycetes and the euascomycetes (filamentous fungi; the subphylum Pezizomycotina of the phylum Ascomycota), *c*. 350 - 400 million years ago, by insertion of a gene encoding allophanate hydrolase into a methylcrotonyl CoA carboxylase (*mccA*) gene, thus creating *DUR1,2 *and inactivating *mccA*. This suggestion was made because of the corresponding dichotomies: the hemiascomycetes have *DUR1,2 *but do not have *mccA *whereas the rest of the fungi have both urease and *mccA *[1]. The present paper investigates the evolutionary origin of *DUR1,2*, the urea amidolyase gene, more thoroughly. We studied the distribution of urea amidolyase, urea carboxylase, and urease proteins in various species across all kingdoms, and biotin-carboxylation domain containing proteins, *i.e*., Acc1, Pyc, PccA, and MccA, in various fungal species. Contrary to our previous speculation, an ancestral urea amidolyase gene likely arose in bacteria and then appeared in the fungal lineage before the divergence of the subphyla Pezizomycotina and Saccharomycotina by prokaryote-to-eukaryote horizontal gene transfer. There have been studies indicating such bacteria-to-fungi horizontal transfers [*e.g.*, [11-15]]. Our study adds yet another important example showing evolutionary interplays between bacteria and fungi and how plastic and opportunistic the fungal genome evolution can be.

## Results and Discussion

### Urea amidolyase is unique to the kingdom fungi among eukaryotes

We have previously shown that long and short forms of urea amidolyase are present in fungi [1]. The urea amidolyase protein of the yeast *Saccharomyces cerevisiae *(phylum Ascomycota; subphylum Saccharomycotina) is 1,835 amino acids (aa) long. As shown in Figure 1, the first 632-aa region in the N-terminus of the protein consists of the amidase domain. The remainder of the sequence is the urea carboxylase domain, which consists of four smaller sub-domains. As mentioned before, the shorter form of urea amidolyase lacks the amidase domain and the urea carboxylase domain exists as a whole protein. This urea carboxylase sequence (1,241 aa) has been identified from a filamentous fungus *Aspergillus nidulans *(phylum Ascomycota; subphylum Pezizomycotina). Using these protein and domain sequences, we first examined if these two forms of urea amidolyase exist in eukaryotes outside of the fungal kingdom.

As shown in Table 1 (see also Additional file 1), urea amidolyase is absent in non-fungal eukaryotic genomes we examined. Blastp similarity search against the NCBI non-redundant (nr) database also showed no sequence similar to urea amidolyase from any other eukaryotic species. However, urea carboxylase and amidase genes are present in all four green algae we examined. In three of the four green algae, the amidase genes are located near the urea carboxylase genes but not adjacent to them. The distance between these two genes ranged from 588 to 6,236 bp in these green algae (see Additional file 2). The absence of urea amidolyase gene but the presence of urea carboxylase and amidase genes in *C. reinhardtii *suggests that the activity of urea amidolyase seen previously in this species [3-5] is not due to the urea amidolyase protein but the combined activity of urea carboxylase and amidase proteins. Although we did not find sequences similar to urea carboxylase from any of the metazoan genomes we examined, similarity search against NCBI nr database turned up two sequences from Hydra (*Hydra magnipapillata*). One of them, however, was found actually to be a sequence of a putative bacterial symbiont. These Hydra sequences are discussed further later. No amidase sequence was found from Hydra or any other eukaryotes other than fungi and green algae.

**Table 1:** Distribution of urea amidolyase and related proteins in eukaryotic species other than fungi.^a^

		**Enzymes**^**b**^			
		
Kingdom	Species	UA	UC	**A**^**c**^	Urease
Plantae (green algae)					
	*Chlamydomonas reinhardtii*	-	1	1^+^	-
	*Volvox carteri*	-	1	1^+^	-
	*Chlorella *sp. NC64A	-	1	1^+^	-
	*Coccomyxa *sp. C-169	-	1	1	-
Plantae (land plants)					
	*Arabidopsis thaliana*	-	-	-	1
	*Oryza sativa*	-	-	-	1^d^
Amoebozoa					
	*Dictyostelium discoideum*	-	-	-	-
Animalia					
	*Nematostella vectensis*	-	-	-	1
	*Drosophila melanogaster*	-	-	-	-
	*Homo sapiens*	-	-	-	-

^a^See Additional file 1 for the sequence sources.

^b^See Figure 1 for the enzyme name abbreviations. The number of sequences found from each genome is shown. '-' indicates that no similar sequence was found.

^c^The amidase gene located close to the urea carboxylase gene (less than 6,250 bp) is indicated with ^+^. See Additional file 2 for the distance between the genes.

^d^Blastp similarity search against the downloaded rice genome showed no sequence similar to urease. However, similarity search against NCBI nr database found a sequence highly similar to urease from *Oryza sativa*.

Urease was found in both plant genomes we examined: *Arabidopsis thaliana *(a dicot) and *Oryza sativa *(a monocot). Similarity search against NCBI nr database also showed a wide distribution of urease in higher plants. While none of the green algal genomes we examined had urease (Table 1), it was identified in distantly related and more ancestral types of green algae (*Ostreococcus *and *Micromonas*) by searching against NCBI nr database. On the other hand, in metazoa, urease was found only in a limited number of genomes. In addition to *Nematostella vectensis *(a sea anemone, Table 1), only three metazoan urease sequences were found in the NCBI nr database (from *Strongylocentrotus purpuratus, Branchiostoma floridae*, and *Ixodes scapularis*). These observations are not consistent with what we observed earlier in fungi, where all fungi that lack urea amidolyase seemed to possess urease ([1]; also described next).

### Distribution of urea amidolyase and other related proteins among fungi

We searched 64 fungal genomes for urea amidolyase, urea carboxylase, and amidase. For selected 27 fungal genomes, we further searched urease as well as proteins that share the biotin-carboxylation and the biotin-lipoyl domains (Acc1, Pyc, MccA, and PccA proteins; see Figure 1). These searches were conducted to examine the earlier hypothesis of Navarathna *et al. *[1] that the fungal urea amidolyase may have been formed by the extension of a biotin carboxylation gene that was already present in fungi.

**Figure 2:**
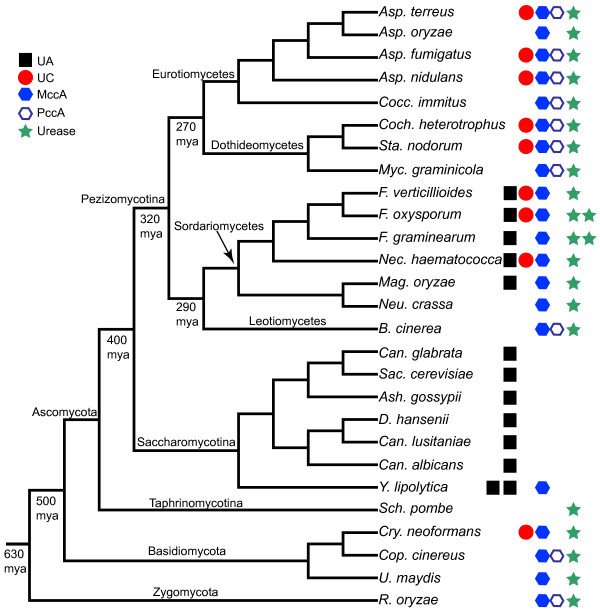
**Distribution of urea amidolyase and related proteins in fungi**. Existence of urea amidolyase and four other proteins are mapped along the current consensus of the fungal phylogeny (summarized from [16,17]). The estimated divergence times (million years ago or mya) are taken from [39]. Refer to Figure 1 for protein name abbreviations. See Table 2 and Additional file 3 for the complete search results.

Our search results are summarized in Table 2 and Additional file 3 (see also Additional files 4 and 5). The results are also mapped on the current consensus of the fungal phylogeny [16,17] in Figure 2. Among the fungi we examined, only the class Sordariomycetes (subphylum Pezizomycotina; except for *Neurospora crassa *and its close relative in the order Sordariales) and the class Saccharomycetes (subphylum Saccharomycotina) had the urea amidolyase sequences. In one species, *Yarrowia lipolytica*, there were two copies of urea amidolyase. Urea carboxylase was found in many but not all of the species in the Pezizomycotina while being completely absent from the Saccharomycotina. Interestingly, except for *Fusarium graminearum *(known also as *Gibberella zeae*), the species belonging to the order Hypocreales (*Nectria, Fusarium*, and *Trichoderma*) had both the urea carboxylase and the urea amidolyase sequences. Many of these species are found in soils and associated with plants [18-20]. Dothideomycetes species did not have urea amidolyase, but many contained amidase as well as urea carboxylase sequences. However, the location of these two genes (amidase and urea carboxylase) was not near each other in their genomes. They were located in different scaffolds or supercontigs (see Additional file 2).

**Table 2:** Distribution of urea amidolyase and related proteins in fungal species.^a^

		**Enzymes**^**c**^					
		
**Taxonomical group**^**b**^	Species	UA	UC	**A**^**d**^	Urease	MccA	PccA
[Zygomycota]	*Rhizopus oryzae*	-	-	-	1	1	1
[Basidiomycota]	*Ustilago maydis*	-	-	-	1	1	-
	*Cryptococcus neoformans*	-	1	-	1	1	-
	*Coprinus cinereus*	-	-	-	1	1	1
[Ascomycota/Taphrinomycotina]							
Schizosaccharomycetes	*Schizosaccharomyces pombe*	-	-	-	1	-	-
[Ascomycota/Pezizomycotina]							
Eurotiomycetes	*Coccidioides immitis*	-	-	-	1	1	1
	*Aspergillus nidulans*	-	1	-	1	1	1
	*Aspergillus fumigatus*	-	1	-	1	1	1
	*Aspergillus terreus*	-	1	-	1	1	1
	*Aspergillus oryzae*	-	-	-	1	1	-
Dothideomycetes	*Mycosphaerella graminicola*	-	-	1	1	1	1
	*Stagonospora nodorum*	-	1	1	1	1	1
	*Cochliobolus heterostrophus*	-	1	1	1	1	1
Leotiomycetes	*Botritis cinerea*	-	-	-	1	1	1
Sordariomycetes	*Neurospora crassa*	-	-	-	1	1	-
	*Magnaporthe oryzae*	1	-	(1)	1	1	-
	*Nectria haematococca*	1	1	(1)	1	1	-
	*Fusarium graminearum*	1	-	(1)	2	1	-
	*Fusarium oxysporum*	1	1	(1)	2	1	-
	*Fusarium verticillioides*	1	1	(1)	1	1	-
[Ascomycota/Saccharomycotina]							
Saccharomycetes	*Yarrowia lipolytica*	2	-	(2)	-	1	-
	*Candida albicans*	1	-	(1)	-	-	-
	*Candida lusitaniae*	1	-	(1)	-	-	-
	*Debaryomyces hansenii*	1	-	(1)	-	-	-
	*Ashbya gossypii*	1	-	(1)	-	-	-
	*Candida glabrata*	1	-	(1)	-	-	-
	*Saccharomyces cerevisiae*	1	-	(1)	-	-	-

^a^See Additional files 4 and 5 for the sequence sources.

^b^The phylum/subphylum (in square brackets) and class are given.

^c^See Figure 1 for the enzyme name abbreviations. The number of sequences found from each genome is shown. '-' indicates that no similar sequence was found.

^d^The amidase sequences that are a part of the urea amidolyase sequences are shown in parentheses.

Consistent with the earlier observation [1], the urease protein was present in all the fungal species examined except for those of the Saccharomycotina. Two species (*F. graminearum *and *F. oxysporum*) had two copies of this protein. Previously only two Sordariomycetes species (*Magnaporthe oryzae*, previously known as *M. grisea*, and *F. graminearum*) were observed to possess both of urease and urea amidolyase. We now confirmed that all Sordariomycetes species except for *N. crassa *and closely related species have both of these enzymes.

Why do the Saccharomycetes species use the energy-dependent, biotin-containing urea amidolyase system and abandon the urease that accomplishes the same overall reaction in a simpler process? This question becomes even more germane when we consider that all strains of *C. albicans *are biotin auxotrophs [21], and it has long been known that 2 to 4 times as much biotin is required for maximum growth of *S. cerevisiae *on urea, allantoic acid, or allantoin as sole nitrogen sources [22]. However, the dichotomy in distribution of urease and urea amidolyase among some fungal lineages coincides precisely with that for the Ni/Co transporter (Nic1p), which is present in those fungi that use urease and absent in those that do not [23]. In Navarathna *et al. *[1], we suggested that the selective advantage of using urea amidolyase over using urease is that it allowed the Saccharomycetes species to jettison all Ni^2+ ^and Co^2+ ^dependent metabolisms and thus to have two fewer transition metals whose concentrations need to be regulated. However, while reasonable for the Saccharomycetes, such selective advantages may not be great enough to abandon the use of urease particularly in the Sordariomycetes species. Further investigation is needed to elucidate whether retaining two types of urea degradation enzymes in the Sordariomycetes species is in fact selectively advantageous rather than redundant.

We also examined the distribution of biotin-carboxylation domain containing enzymes. Acc1 and Pyc were present in all the fungal species we examined. MccA was absent almost completely from the Saccharomycetes and *Schizosaccharomyces pombe *(phylum Ascomycota; subphylum Taphrinomycotina), but was present in the rest of the fungi we examined. PccA was present in fewer species than MccA was, and was completely absent from the classes Saccharomycetes and Sordariomycetes. MccA was present along with urea amidolyase and urea carboxylase in three species (*Fusarium verticilloides, F. oxysporum*, and *Nectria haematococca*), and along with only urea amidolyase in three other species (*F. graminearum, M. oryzae*, and *Y. lipolytica*). A phylogenetic analysis using the biotin-carboxylation domains of Pyc, Acc1, MccA, PccA, urea amidolyase, and urea carboxylase from fungi showed that these domain sequences were highly diverged. Bootstrap analysis did not show any significantly supported clustering of urea amidolyase and urea carboxylase with any of the other four enzymes (see Additional file 6). Urea amidolyase and urea carboxylase appear to have no clear direct origin among the other biotin-carboxylation domain containing proteins. Or such divergence may have happened such a long time ago that we can no longer identify the origin.

### Distribution of urea amidolyase and other related proteins among eubacteria

In order to elucidate further the origin of long and short forms of urea amidolyase found in fungi: whether they share a common evolutionary origin or arose independently, we performed extensive similarity searches using these protein and domain sequences among 56 bacterial genomes. As shown in Table 3 (see also Additional file 7), the longer form of urea amidolyase (~1,800 aa) was found only in one bacterium, *Pantoea ananatis *(class Gammaproteobacteria). This bacterium, which previously belonged to the genus *Erwinia *but was recently reclassified into the genus *Pantoea*, is a well-known plant pathogen with a reported case of it also being a human-pathogen [24,25]. This bacterium and its related species are usually isolated from soil, fruits, and vegetables [24]. Urea carboxylase (~1,200 aa), the shorter form of urea amidolyase, was found in bacterial species scattered among a wide range of groups. Almost all bacteria with urea carboxylase also had amidase. These two enzymes are encoded in two different genes in bacteria, but are located next to each other in most of the bacterial genomes we examined (see Additional file 8). In two species (*Wolinella succinogenes*, class Epsilonproteobacteria; and *Gloeobacter violaceus*, phylum Cyanobacteria), the two genes were not adjacent to each other but only 943 bp and 1,701 bp apart, respectively, while in another Cyanobacteria species (*Cyanothece *sp.), the two genes were located far apart (979,743 bp). *Sorangium cellulosum *(class Deltaproteobacteria) and *Nitrosomonas europaea *(class Alphaproteobacteria) had urea carboxylase but lacked amidase. Three Gammaproteobacteria species have two urea carboxylase genes, only one of which lies next to the amidase gene. *P. ananatis*, a gammaproteobacteria, which has urea amidolyase (the long form), also has urea carboxylase (the short form). Furthermore, *P. ananatis *has no independent amidase gene. The only amidase sequence present in this bacterium is the domain of the urea amidolyase gene. It seems reasonably likely that fusion of the amidase and urea carboxylase genes occurred in *P. ananatis *to generate the long form of the urea amidolyase gene similar to those found in fungi.

**Table 3:** Distribution of urea amidolyase and related proteins in eubacterial species.^a^

		**Enzymes**^**b**^			
		
Phylum or Class	Species	UA	UC	**A**^**c**^	**Urease**^**d**^
Alphaproteobacteria	*Caulobacter crescentus NA1000*	-	1	1*	-
	*Asticcacaulis excentricus CB 48*	-	1	1*	-
	*Sinorhizobium medicae WSM419*	-	-	-	α,β,γ
Betaproteobacteria	*Achromobacter piechaudii ATCC 43553*	-	1	1*	-
	*Bordetella pertussis Tohama I*	-	-	-	α,β,γ
	*Nitrosomonas europaea ATCC 19718*	-	1	-	-
	*Neisseria meningitidis FAM18*	-	-	-	-
	*Burkholderia sp. CCGE1001*	-	1	1*	α,β,γ
Gammaproteobacteria	*Escherichia coli O111:H- str. 11128*	-	-	-	α,β,γ
	*Yersinia pestis Angola*	-	-	-	α,β
	*Haemophilus influenzae 86-028NP*	-	-	-	α,β,γ
	*Pantoea ananatis LMG 20103*	1	1	(1)	-
	*Pantoea sp. At-9b*	-	2	1*	-
	*Shewanella oneidensis MR-1*	-	-	-	-
	*Pseudomonas aeruginosa LESB58*	-	-	-	α,β,γ
	*Coxiella burnetii Dugway 5J108-111*	-	-	-	-
	*Pectobacterium carotovorum subsp. carotovorum PC1*	-	2	1*	-
	*Xanthomonas campestris pv. campestris str. B100*	-	-	-	-
	*Cellvibrio japonicus Ueda107*	-	2	1*	-
	*Teredinibacter turnerae T7901*	-	1	1*	α,β,γ
	*Marinomonas sp. MED121*	-	1	1*	α,γ
	*Klebsiella pneumoniae 342*	-	1	1*	α,β,γ
	*Pseudomonas fluorescens SBW25*	-	-	-	α,β,γ
Deltaproteobacteria	*Geobacter sp. M21*	-	-	-	-
	*Sorangium cellulosum 'So ce 56'*	-	1	-	α,β/γ
Epsilonproteobacteria	*Helicobacter pylori B38*	-	-	-	α,β/γ
	*Wolinella succinogenes DSM 1740*	-	1	1^+^	-
Acidobacteria	*Acidobacterium capsulatum ATCC 51196*	-	-	-	-
	*Solibacter usitatus Ellin6076*	-	1	1*	-
Cyanobacteria	*Synechococcus sp. PCC 7002*	-	-	-	α,β,γ
	*Gloeobacter violaceus PCC 7421*	-	1	1^+^	-
	*Cyanothece sp. PCC 7425*	-	1	1	α,β,γ
Deinococcus-Thermus	*Thermus thermophilus HB8*	-	-	-	-
	*Deinococcus deserti VCD115*	-	-	-	-
Chloroflexi	*Dehalococcoides ethenogenes 195*	-	-	-	-
Aquificae	*Aquifex aeolicus VF5*	-	-	-	-
Thermotogae	*Thermotoga maritima MSB8*	-	-	-	-
Fusobacteria	*Fusobacterium nucleatum subsp. Nucleatum ATCC 25586*	-	-	-	-
Verrucomicrobia	*Verrucomicrobium spinosum DSM 4136*	-	1	1*	α,β,γ
Chlamydiae	*Chlamydophila pneumoniae CWL029*	-	-	-	-
	*Chlamydia trachomatis B/TZ1A828/OT*	-	-	-	-
Bacterioidetes	*Porphyromonas gingivalis W83*	-	-	-	-
Chlorobi	*Chlorobium limicola DSM 245*	-	-	-	-
Fibrobacteres	*Fibrobacter succinogenes subsp. succinogenes S85*	-	-	-	-
Actinobacteria	*Mycobacterium tuberculosis F11*	-	-	-	α,β,γ
	*Corynebacterium aurimucosum ATCC 700975*	-	-	-	-
	*Streptomyces avermitilis MA-4680*	-	1	1*	α,β,γ; α,β/γ
	*Bifidobacterium longum subsp. infantis ATCC 15697*	-	-	-	α,β/γ
Spirochaetes	*Borrelia burgdorferi ZS7*	-	-	-	-
	*Treponema denticola ATCC 35405*	-	-	-	-
Planctomycetes	*Rhodopirellula baltica SH 1*	-	-	-	-
Firmicutes	*Clostridium botulinum A2 str. Kyoto*	-	-	-	-
	*Mycoplasma hyopneumoniae 7448*	-	-	-	-
	*Streptococcus pneumoniae 70585*	-	-	-	-
	*Bacillus anthracis str. CDC 684*	-	-	-	-
	*Roseburia intestinalis L1-82*	-	1	1*	-

^a^See Additional file 7 for the sequence sources.

^b^See Figure 1 for the enzyme name abbreviations. The number of sequences found from each genome is shown. '-' indicates that no similar sequence was found.

^c^The amidase gene located next to (within 200 bp) the urea carboxylase gene is indicated with *. The amidase gene located close to (within 6,500 bp) but not next to the urea carboxylase gene is indicated with ^+^. See Additional file 8 for the distance between the genes. The amidase sequences that are a part of the urea amidolyase sequences are shown in parentheses.

^d^For urease, the search results for three subunits (α, β, or γ) are shown. β/γ indicates that the β and γ subunits are fused into one gene.

The urease protein in bacteria occurs as a trimer of alpha, beta, and gamma subunits encoded by separate genes forming a gene cluster, whereas in eukaryotes a single gene encodes the urease protein, a fused protein representing the three bacterial subunits [26]. In some bacteria, beta and gamma subunits are fused and encoded by one gene (denoted with ß/γ in Table 3) while in others either beta- or gamma-subunit gene was missing. As shown in Table 3, existence of these urease-subunit genes was scattered throughout the bacterial groups. Of 56 bacterial genomes we examined, 31 had either or both of urease and amidase/urea carboxylase (or urea amidolyase). Only seven of 31 bacterial species had all three genes. Consistent with what we observed in fungi, there appears to be a certain degree of dichotomy in possession of urease genes or amidase/urea carboxylase (or urea amidolyase) genes among bacterial genomes.

### Phylogenetic analysis of amidase domain sequences

In order to elucidate the evolutionary origin of eukaryotic urea amidolyase proteins, we performed phylogenetic analysis among amidase, urea amidolyase, and urea carboxylase identified across kingdoms. Phylogenies were reconstructed using amidase and urea carboxylase sequences separately.

**Figure 3:**
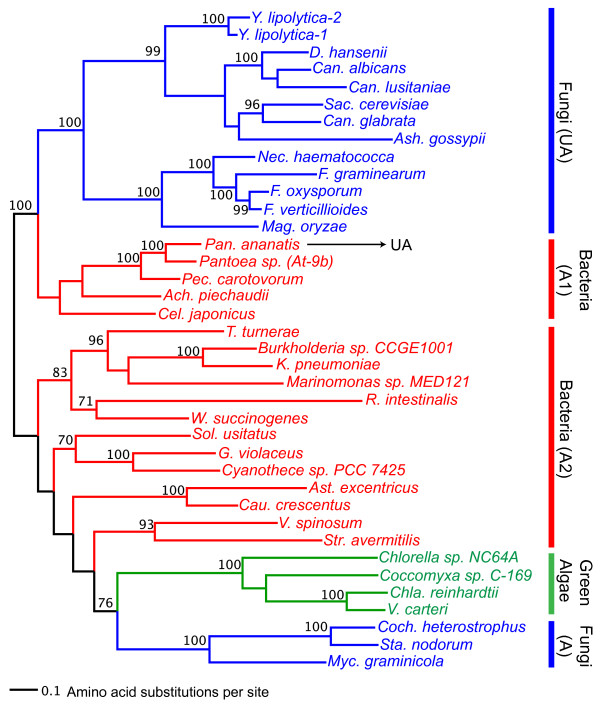
**Maximum-likelihood phylogeny of amidase protein sequences**. The maximum-likelihood phylogeny was reconstructed using the protein sequences from the amidase domains of the urea amidolyase proteins and the amidase proteins. The numbers above or below the internal branches show bootstrap values (%). Only bootstrap values equal to or higher than 70% are shown. Branches are colored as follows: blue for fungi, green for green algae, and red for bacteria. The bacterial urea carboxylase forms two separate groups denoted by A1 and A2. See Additional files 1, 4, and 7 for the sequence sources.

Figure 3 is the maximum-likelihood phylogenetic tree reconstructed from the amidase domain sequences from urea amidolyase and the amidase protein sequences from fungi, green algae, and bacteria (the minimum-evolution tree is shown in Additional file 9). It shows that the fungal amidase domain from urea amidolyase (shown in blue and denoted by UA in Figure 3), and the stand-alone fungal amidase protein that exists on its own (shown in blue and denoted by A in Figure 3) cluster separately, implying that they have evolved independently. The amidase sequences from green algae (shown in green in Figure 3) cluster with the stand-alone amidase protein from fungi, however, with not very strong bootstrap support (76%).

Bacterial amidase sequences also cluster into two groups (shown in red in Figure 3). Amidases from four gammaproteobacteria species (*P. ananatis, Pantoea sp. At-9b, Pectobacterium carotovorum*, and *Cellvibrio japonicus*) and one betaproteobacteria species (*Achromobacter piechaudii*) form a cluster (denoted by A1 in Figure 3). Notably, the amidase sequence of *P. ananatis *is part of the urea amidolyase, and the amidase genes of the other three gammaproteobacteria species lie immediately adjacent to their urea carboxylase genes (see Additional file 8). These bacterial amidases cluster with fungal amidases from urea amidolyase with a strong bootstrap support (100%). Compared to the fungal stand-alone amidases (Fungi A), the fungal amidase-domain sequences from urea amidolyase (Fungi UA) are clearly more closely related to the bacterial amidases, especially to those from *P. ananatis *and a small number of gamma- and betaproteobacteria species (Bacteria A1).

### Phylogenetic analysis of urea carboxylase domain sequences

**Figure 4:**
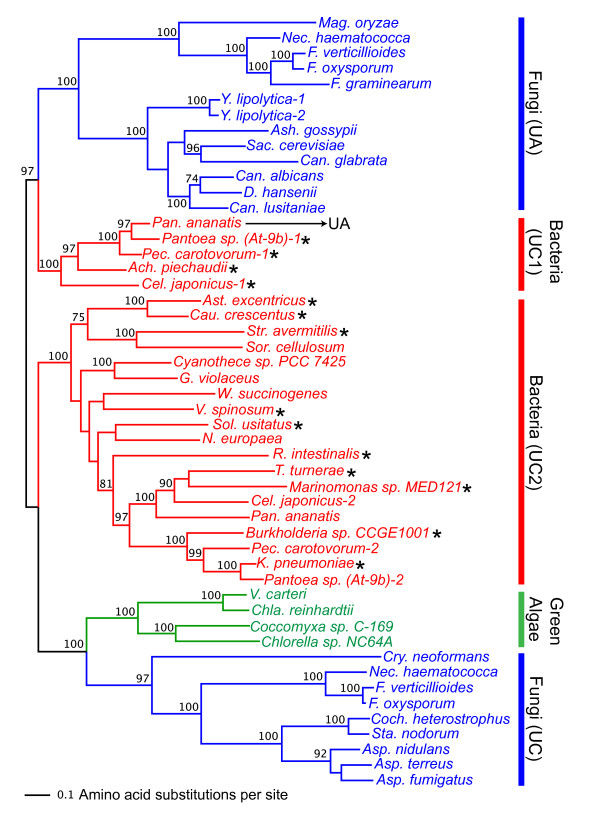
**Maximum-likelihood phylogeny of urea carboxylase protein sequences**. The maximum-likelihood phylogeny was reconstructed using the protein sequences from the urea carboxylase domains of the urea amidolyase proteins and the urea carboxylase proteins. The numbers above or below the internal branches show bootstrap values (%). Only bootstrap values equal to or higher than 70% are shown. Branches are colored as follows: blue for fungi, green for green algae, and red for bacteria. The bacterial urea carboxylase forms two separate groups denoted by UC1 and UC2. The asterisks beside the bacterial names indicate that their urea carboxylase genes are adjacent to the amidase genes in their genomes. See Additional file 8 for the distance between these genes. See Additional files 1, 4, and 7 for the sequence sources.

Figure 4 shows the result of maximum-likelihood phylogenetic analysis using the urea carboxylase protein and urea carboxylase domain sequences from urea amidolyase (the minimum-evolution tree is shown in Additional file 10). The urea carboxylase sequence (~1,200 aa) is twice longer than the amidase sequence (~600 aa), which resulted in a better resolution in the reconstructed phylogeny. Bacterial urea carboxylase sequences were clearly divided into two clusters (denoted by UC1 and UC2 in Figure 4) where both were supported by 100% bootstrap values. The UC1 group, which consists of the five species of gamma- and betaproteobacteria (*P. ananatis, Pantoea At-9b, P. carotovorum, C. japonicus*, and *A piechaudii*), clustered closely with the fungal urea amidolyase (UA) with a high bootstrap value (97%). These five bacterial species are the same five species found in Figure 3 (A1) whose amidases clustered with the amidase-domain sequences of the fungal urea amidolyase. Four of these five bacterial species have a second urea carboxylase gene. Thus, the duplication event that created these two sets of urea carboxylase genes must have happened before the divergence of the five proteobacteria. Based on the deep divergence between the paralogous groups (UC1 and UC2) and the somewhat slower evolution observed in UC1 (the urea carboxylase genes found only in five gamma/betaproteobacteria species), we speculate that the close functional association with amidase likely arose in the UC1 group to create a fused single gene, urea amidolyase, in *P. ananatis*, and thus changed the evolutionary rate and pattern in this copy of urea carboxylase.

We also see two separate and strongly supported clusters of urea carboxylase sequences in fungi. One cluster is of the urea carboxylase domain from urea amidolyase (UA, 100% bootstrap support) whereas the other cluster is of the urea carboxylase protein sequence (UC, 97% bootstrap support). It shows that the urea carboxylase sequences in the two groups have independently evolved over a long period of time. Since urea carboxylase was found in the phylum Basidiomycota (represented by *Cryptococcus neoformans *in Figure 4) and it clustered with other urea carboxylase proteins, the divergence between urea carboxylase and urea amidolyase in fungi must have preceded the Ascomycota-Basidiomycota divergence. As we discuss in the next section, the formation of urea amidolyase with acquisition of the amidase domain seems to have happened most likely in a bacterial lineage. Note that the urea carboxylases from green algae clustered with the fungal urea carboxylases (with 100% bootstrap support) rather than with the fungal urea amidolyases. This clustering pattern is consistent with what we observed in the amidase phylogeny (Figure 3) where green algal genes clustered with the stand-alone version of the fungal amidase genes rather than with the amidase-domain sequence of urea amidolyase. Although in some green algae, amidase and urea carboxylase genes are located relatively closely (within 588 to 6,236 bp; Additional file 2), their evolution is completely independent from urea amidolyase genes found in fungi.

As mentioned before, two Hydra urea carboxylase sequences were found from the NCBI nr database search. One of them was actually found to be a sequence of a putative bacterial symbiont, *Curvibacter *(betaproteobacteria) (described in NCBI gi|260221606 entry). Phylogenetic analysis clearly showed that this sequence belongs to the bacterial urea carboxylase (UC2) group (see Additional file 11). The other Hydra sequence clustered with urea carboxylase sequences from green algae and fungi (93% bootstrap support).

### Bacterial origins of the fungal urea amidolyase and urea carboxylase

Our phylogenetic analysis did not support the previous hypothesis that the fungal urea amidolyase and urea carboxylase sequences are formed from fungal biotin-carboxylation domain containing proteins such as MccA or PccA. Instead, our conclusion is that the urea amidolyase and urea carboxylase genes currently found in fungi and green algae, as well as in Hydra, are the results of horizontal gene transfer events from bacteria. This is based on observations such as the abundant distribution of the shorter form of urea amidolyase, *i.e.*, urea carboxylase, as well as the single occurrence so far of urea amidolyase (the long form) in bacteria, coupled with the rarity of both forms of urea amidolyase in eukaryotes except in the fungal kingdom, in some green algae, and in Hydra.

Phylogenetic analysis of amidase and urea carboxylase sequences across kingdoms showed that the urea carboxylase domain in urea amidolyase and the urea carboxylase protein itself have undergone extensive independent evolution. Fungal urea amidolyase proteins are more closely related to one of the two groups of bacterial urea carboxylase. Furthermore, one of these bacteria (*P. ananatis*) has a unique urea amidolyase gene, a product of amidase/urea carboxylase gene fusion. The direction of the horizontal gene transfer seems to be from a bacterial lineage to a fungal lineage, since in bacteria other than *P. ananatis*, urea carboxylase and amidase exist as two independent genes although they are located next to each other. Inspection of introns in fungal urea amidolyase genes corroborates this hypothesis further. Fungal urea amidolyases are either single or double-exon genes (see Additional file 2). All Saccharomycetes species except for *Y. lipolytica *have single-exon urea amidolyase genes. While in the three Sordariomyetes species (*M. oryzae, N. haematococca*, and *F. graminearum*) the single intron was inserted towards the end of the urea carboxylase domain, in the duplicated *Y. lipolytica *genes the single intron was inserted at the beginning of the amidase domain. These observations indicate that the introns in these fungal urea amidolyase genes must have been acquired independently during their evolution as fungal genes. Therefore, fusion of the two genes appears to have happened in the ancestral bacterial species close to *P. ananatis*, and this fused gene must have been transferred to a fungal lineage.

Since so far we found the urea amidolyase protein only in one bacterial species, it is probable that the fusion of urea carboxylase and amidase to form bacterial urea amidolyase is a recent event specific to this bacterial lineage. If this is the case, the fusion event in *P. ananatis *could be also independent from those that produced fungal urea amidolyases. However, we did not find any unfused fungal urea carboxylase sequences clustered with urea amidolyase in our phylogenetic analysis (Figure 4), nor did we find any unfused fungal amidase sequences clustered with urea amidolyase (Figure 3). Therefore, if the fusion happened in fungal lineage, it must have happened soon after the two bacterial genes (amidase and urea carboxylase) were acquired by an ancestral fungal species. Regardless of the timing of the fusion event, association between the amidase and urea carboxylase sequences for the urea amidolyase function and subsequent divergence of these sequences from the other paralogous set must have started in bacterial lineage.

Compared to urea amidolyase, urea carboxylase genes in fungi have a wider range in the number of exons, 1-16 exons, implying again their independent evolution as well as a greater number of accumulated changes. Note that the single introns found in the urea carboxylase genes of *N. haematococca *and *F. oxysporum *are both at the beginning of the genes and of similar lengths (55-56 bp; see Additional file 2). It indicates that the common ancestor of these species acquired a single intron in the urea carboxylase gene and it happened independently from the intron acquisition in *N. haematococca *urea amidolyase. Interestingly, the number of introns in urea carboxylase and amidase genes in green algae is much higher than the number of introns in the fungal orthologues. This is in agreement with the observation that the *Chlamydomonas reinhardtii *genome has much higher percentage of genes with introns and a much greater number of exons per gene (88% and 7.4) as compared to *S. cerevisiae *(5% and 1) and *S. pombe *(43% and 2) [27].

There have been studies presenting cases of bacteria-to-fungi horizontal gene transfers. For example, Hall *et al. *[11] found ten potential such cases in *S. cerevisiae *and one in *Ashbya gossypii*. Fitzpatrick *et al. *[12] reported two *Candida parapsilosis* genes as bacterial origin. Garcia-Vallvé *et al. *[13] showed that many glycosyl hydrolase genes in the rumen fungus *Orpinomyces joyonii *were acquired from bacteria. Schmitt and Lumbsch [14] showed that the polyketide synthase in lichen-forming fungi were results of ancient horizontal gene transfer from Actinobacteria. A recent study, the largest of its kind, by Marcet-Houben and Gabaldón [15] detected 713 transferred genes in 60 fungal genomes. Therefore, horizontal gene transfers from bacteria to fungi do not appear to be rare events. We identified yet another such example.

### Proposed model for the urea carboxylase and urea amidolyase evolution

**Figure 5:**
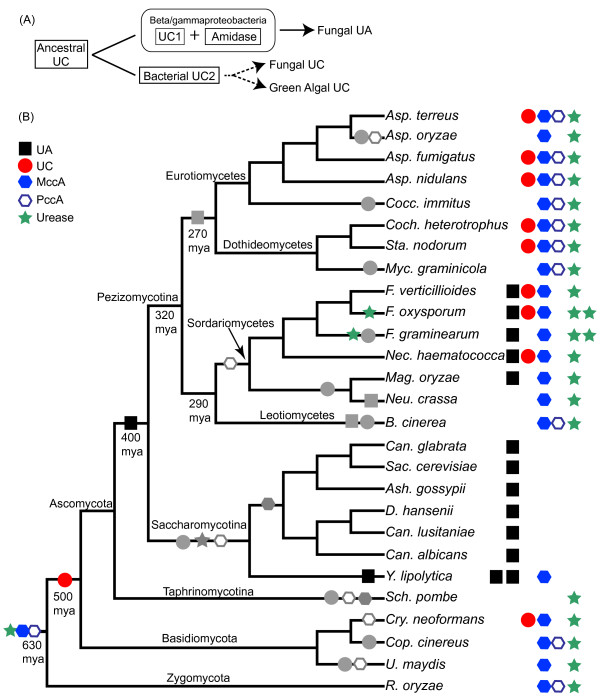
**Evolutionary model of urea carboxylase and urea amidolyase in fungi**. (A) The evolution of the two types of bacterial urea carboxylases, UC1 and UC2, and the subsequent transfer of those genes to fungi and green algae. The arrows represent possible horizontal gene-transfer events. Dashed arrows indicate that both horizontal transfer and vertical transmission are possible. (B) Acquisition and loss events of the urea amidolyase and related proteins inferred within fungal evolution. The fungal consensus phylogeny and the presence/absence table for five proteins are the same as Figure 2. Within the tree, the colored symbols indicate gene-acquisition events while the grey symbols indicate the deletion of that gene.

Figure 5 illustrates our proposed model for the evolution of urea carboxylase and urea amidolyase genes in fungi. As presented in Figure 5A, an ancestral urea carboxylase sequence in bacteria duplicated in the beta/gammaproteobacteria lineage and evolved into two genes (UC1 and UC2). Since in many bacterial genomes, urea carboxylase and amidase genes are located adjacent to each other (see Additional file 8), it is plausible that before the duplication, the ancestral urea carboxylase gene already had an associated function with the amidase gene. However, the creation of duplicated redundant copies of the urea carboxylase gene in beta/gammaproteobacteria species appears to have reinforced the association between the two genes and changed their evolutionary pattern and rate in these bacteria. This amidase-associated copy of bacterial urea carboxylase gene (UC1) was subsequently fused with the amidase gene to form a single urea amidolyase gene. The fused gene was later transferred to an ancestral ascomycete lineage before the divergence of the Pezizomycotina and Saccharomycotina. Alternatively, the gene fusion could have happened in an ancestral fungal species soon after the region containing amidase and urea carboxylase genes was transferred from bacteria.

The other bacterial urea carboxylase gene (UC2) may have also been acquired by fungi, green algae, as well as Hydra. Since our phylogenetic analysis did not show independent origins for these urea carboxylase genes, the acquisition of this enzyme into fungi, green algae, and Hydra must have happened around the time of divergence among these groups of organisms. It may have been by a single event, likely before the divergence of these organisms. Then we cannot eliminate the possibility that what we observed in the urea carboxylase genes is the result of simple vertical evolution from bacteria to eukaryotes. Either way, however, many eukaryotes including the entire metazoa and land plants must have lost these genes. As we mentioned before (and shown also in Figure 5B), even within fungi, the urea carboxylase gene is not retained in many species. Considering that either scenario requires such a high number of loss events, there would be other possible scenarios. One group of organisms (either green algae, Hydra, or fungi) may have acquired a urea carboxylase gene from bacteria first. Later this gene may have been transferred to other organisms. Although this scenario requires fewer loss events, the main question is how such horizontal gene transfers can happen between green algae, Hydra, and fungi, or among any of their ancestral organisms.

In fungi, the introduction of the urea carboxylase gene happened earlier than that of the urea amidolyase gene as shown in Figure 5B. The urea carboxylase gene (red circle) was acquired in fungi before the divergence of the phyla Ascomycota and Basidiomycota. The acquisition could have been after the divergence of the phylum Zygomycota or alternatively the gene was lost from the Zygomycota lineage. Some Basidiomycota species subsequently lost the gene (the lost events are indicated with grey symbols in Figure 5B). In the phylum Ascomycota, this gene was again lost in the subphyla Taphrinomycotina (it includes *S. pombe*) and Saccharomycotina. Further losses of this gene happened in some species of the subphylum Pezizomycotina. The urea carboxylase gene appears to become easily dispensable in many species, which may be related to the genomic and metabolic environment of the organisms. The same seems to be the case with MccA and PccA. The introduction of the urea amidolyase gene (black square) in fungi took place before the divergence of the subphyla Pezizomycotina and Saccharomycotina but probably after the divergence of the subphylum Taphrinomycotina (at least after the phylum Ascomycota diverged from the ancestral lineage). Within the subphylum Pezizomycotina, the urea amidolyase gene was lost in many groups but retained in almost all species in the class Sordariomycetes (absent in the order Sordariales species). The urea amidolyase gene was retained in all Saccharomycotina species, and even recently duplicated in *Y. lipolytica*.

## Conclusions

We have presented a possible scenario of horizontal gene transfer of the urea amidolyase and urea carboxylase genes from bacteria to fungi. Plastic and opportunistic genome evolution in bacteria and fungi and their evolutionary interplay must have allowed the Saccharomycetes fungi to abandon the use of nickel-containing urease. It contributed to optimizing these organisms toward Ni^2+ ^(and Co^2+^)-independent cellular metabolisms. Further detailed studies of a wider range of gene families would reveal the importance of acquisition of bacterial genes in fungal evolution.

## Methods

### Similarity searches

Similarity searches for protein sequences were performed using blastp (version 2.2.17 [28]). For urea amidolyase search, the *S. cerevisiae *sequence (P32528) was used as a query. Search was performed using both the full sequence as well as only the amidase domain of this sequence. To search for urea carboxylase sequences, *A. nidulans *sequence (P38095) was used as a query. To search for other urea carboxylase domain containing proteins, the *S. cerevisiae *Acc1 (Q00955) and Pyc (P11154), *A. nidulans *MccA (Q6T5L7), and *Aspergillus *related *Neosartorya fischeri *PccA (A1DF70) were used as query sequences. The urease sequence from *A. fumigatus *(Q6A3P9) was used as a query sequence to search for urease.

We performed these searches against 56 bacterial genomes, 64 fungal genomes, and 10 non-fungal eukaryotic genomes (including 4 green algae, 2 land plants, 1 amoebozoa, and 3 animals). The species names, taxonomical groups, and the sources of the sequences are listed in Additional files 1, 4, 5, and 7. The species were chosen such that all major bacterial, fungal, and other eukaryotic groups were represented from a tree of life [*e.g.*, [29]]. For fungi, preliminary search for urea amidolyase, urea carboxylase, and amidase was done in 64 genomes and further analysis was done using 27 selected fungal genomes (noted with * in Additional file 3). The non-redundant (nr) database at National Center for Biotechnology Information (NCBI) was also searched for urea amidolyase, urea carboxylase, and urease protein sequences using blastp.

All protein sequences were highly conserved, and similar sequences were clearly identifiable in the results obtained by blastp similarity search. The E-value threshold for each protein hit was as follows: 1 × 10^-49 ^for amidase, 0 for urea amidolyase and urea carboxylase, 1 × 10^-12 ^for urease, 1 × 10^-111 ^for MccA, 1 × 10^-115 ^for PccA, and 0 for Pyc and Acc1. The default parameters were used with blastp program (version 2.2.17), which include BLOSUM62 scoring matrix, low-complexity filtering, gap-open and gap-extend penalties of 11 and 1, respectively. In order to obtain the E-values comparable among different genome sizes, the "effective length of database" was set to 500,000,000 (using -z option). This also makes the E-values obtained from each genome search equivalent to those obtained against NCBI nr database.

### Multiple alignment and phylogenetic analysis

Multiple alignments of protein sequences were generated using MAFFT (version 6.240 [30]) with default parameters (FFT-NS-2, a progressive FFT alignment with two tree-building cycles). The maximum-likelihood phylogeny [31] was reconstructed as implemented in raxmlHPC-MPI (version 7.0.4 [32]) using the following options: '-m PROTMIXWAG' to use WAG amino-acid substitution model [33] with a fixed number approximation followed by a refined gamma-model of rate heterogeneity, '-f a' for a rapid bootstrap analysis, '-x 1234' to set the random seed, and '-# 1000' for 1000 pseudoreplicates for bootstrap analysis. To gather the bootstrap values, the 'consense' program of the Phylip package (v. 3.68 [34]) was used. The minimum-evolution phylogeny [35] was reconstructed as implemented in MEGA4 [36] using the JTT amino-acid substitution model [37] with 1000 pseudoreplicates for bootstrap analysis.

## Authors' contributions

PKS collected data, carried out all the analyses, and drafted the manuscript. KWN and SDH conceived of the study, contributed the discussion, and revised the manuscript. ENM supervised the entire process of the study, contributed the discussion, and revised the manuscript. All authors read and approved the final manuscript.

## Supplementary Material

Additional file 1 **Sequence sources for the non-fungal eukaryotic sequences used in this study**. Click here for file

Additional file 2 **Number of exons in urea amidolyase and related genes and their distance in eukaryotic genomes**. Click here for file

Additional file 3 **Distribution of urea amidolyase, urea carboxylase, and amidase proteins in 64 fungal species**. Click here for file

Additional file 4 **Sequence sources of the urea amidolyase, urea carboxylase, and amidase from 64 fungal species**. Click here for file

Additional file 5 **Sequence sources of the urease, methylcrotonoyl-CoA carboxylase, and propionyl-CoA carboxylase from the selected 27 fungal species**. Click here for file

Additional file 6 **Maximum-likelihood phylogeny of the carboxylation-domain sequences from urea carboxylase, urea amidolyase, methylcrotonoyl-CoA carboxylase, propionyl-CoA carboxylase, pyruvate carboxylase, and acetyl-CoA carboxylase**. Click here for file

Additional file 7 **Sequence sources of urea amidolyase, urea carboxylase, and amidase in eubacterial genomes**. Click here for file

Additional file 8 **Distance between amidase and urea carboxylase genes in eubacterial genomes**. Click here for file

Additional file 9 **Minimum-evolution phylogeny of amidase protein sequences**. Click here for file

Additional file 10 **Minimum evolution phylogeny of urea carboxylase protein sequences**. Click here for file

Additional file 11 **Maximum-likelihood phylogeny of urea carboxylase protein sequences including the two Hydra sequences**. Click here for file
