# Supplementary material for: Molecular evolution of urea amidolyase and urea carboxylase in fungi
Source: BMC Evol Biol. 2011 Mar 29;11:80. doi: 10.1186/1471-2148-11-80 (PMC3073912; doi:10.1186/1471-2148-11-80)
Supplement: Additional file 1 — Sequence sources for the non-fungal eukaryotic sequences used in this study. [file 1471-2148-11-80-S1.PDF]

Table S1. Sequence sources for the non-fungal eukaryotic sequences used in this study.

Kingdom		Enzymes[b]			
Species [genome ver. or Acc #]	Source[a]	UA	UC	A	Urease
Plantae (green algae)					
Chlamydomonas reinhardtii [v3.1]	JGI	-	133000	196482	-
Volvox carteri f. nagariensis [v1.0]	JGI	-	98356	98357	-
Chlorella sp. NC64A [v1.0]	JGI	-	133810	57824	-
Coccomyxa sp. C-169 [v2.0]	JGI	-	19857	30676	-
Plantae (land plants)					
Arabidopsis thaliana [NC_003070, NC_003071, NC_003074, NC_003075, NC_003076]	NCBI	-	-	-	15220459
Oryza sativa v6.1	Rice Genome	-	-	-	-
Amoebozoa					
Dictyostelium discoideum	DictyBase	-	-	-	-
Animalia					
Nematostella vectensis [v1.0]	JGI	-	-	-	98292
Drosophila melanogaster [rel 5.12]	Flybase	-	-	-	-
Homo sapiens	UniProtKB	-	-	-	-

[a]JGI: Joint Genome Institute (http://www.jgi.doe.gov), Rice Genome: Rice Genome Annotation Project (http://rice.plantbiology.msu.edu/), NCBI: National Center for Biotechnology Information (http://www.ncbi.nlm.nih.gov/), DictyBase: *Dictyostelium discoideum* database (http://dictybase.org/), Flybase: A Database of *Drosophila* Genes & Genomes (http://flybase.org/), and UniProtKB: The UniProt Knowledgebase (http://www.uniprot.org/).

[b]See Figure 1 for the enzyme name abbreviations. The IDs of sequences found from each genome are shown. '-' indicates that no similar sequence was found.
