# Supplementary material for: Molecular evolution of urea amidolyase and urea carboxylase in fungi
Source: BMC Evol Biol. 2011 Mar 29;11:80. doi: 10.1186/1471-2148-11-80 (PMC3073912; doi:10.1186/1471-2148-11-80)
Supplement: Additional file 2 — Number of exons in urea amidolyase and related genes and their distance in eukaryotic genomes. [file 1471-2148-11-80-S2.PDF]

**Table S2. Number of exons in urea amidolyase and related genes and their distance in eukaryotic genomes.**

| Kingdom / Species | No. of exons | | | No. of bp between UC and A[b] |
|---|---|---|---|---|
| | UA[a] | UC[a] | A[a] | |
| Plantae (green algae) | | | | |
| *Chlamydomonas reinhardtii* v3.1 | | 23 | 9 | 1,567 (0) |
| *Volvox carteri f. nagariensis* v1.0 | | 25 | 6 | 588 (0) |
| *Chlorella* sp. NC64A v1.0 | | 32 | 14 | 6,236 (3) |
| *Coccomyxa* sp. C-169 v2.0 | | 25 | 9 | Scaffolds 15 and 20 |
| Fungi | | | | |
| *Cryptococcus neoformans* H99 | | 16 | | |
| *Aspergillus nidulans* FGSC A4 | | 8 | | |
| *Aspergillus fumigatus* Af293 | | 8 | | |
| *Aspergillus terreus* NIH2624 | | 7 | | |
| *Mycosphaerella graminicola* v2.0 | | | 1 | |
| *Stagonospora nodorum* SN15 | | 3 | 2 | Supercontigs 3 and 12 |
| *Cochliobolus heterostrophus* C5 | | 4 | 1 | Scaffolds 1 and 8 |
| *Magnaporthe oryzae* ATCC 64411 | 2[c] | | | |
| *Nectria haematococca* v2.0 | 2[c] | 2[e] | | |
| *Fusarium graminearum* PH-1 (NRRL 31084) | 2[c] | | | |
| *Fusarium oxysporum* 4286 | 1 | 2[e] | | |
| *Fusarium verticillioides* 7600 | 1 | 1 | | |
| *Yarrowia lipolytica* CLIB122 | 2[d], 2[d] | | | |
| *Candida albicans* SC5314 | 1 | | | |
| *Candida lusitaniae* ATCC 42720 | 1 | | | |
| *Debaryomyces hansenii* CBS767 | 1 | | | |
| *Ashbya gossypii* ATCC 10895 | 1 | | | |
| *Candida glabrata* CBS138 | 1 | | | |
| *Saccharomyces cerevisiae* S288C | 1 | | | |

[a]See Figure 1 for the enzyme name abbreviations.

[b]The number of genes present between UC and A genes are given in parentheses. When exact distance between the two genes is not known, the locations of the two genes are given.

[c]In these genes, the intron is located towards the end of the urea-carboxylase domain.

[d]In these genes, the intron is located at the beginning of the amidase domain.

[e]In these genes, the intron is located at the beginning of the urea carboxylase sequence. The lengths of the intron and second exon in *N. haematococca* (56 bp and 3,502 bp) is similar to those in *F. oxysporum* (55 bp and 3,499 bp).
