# Supplementary material for: Molecular evolution of urea amidolyase and urea carboxylase in fungi
Source: BMC Evol Biol. 2011 Mar 29;11:80. doi: 10.1186/1471-2148-11-80 (PMC3073912; doi:10.1186/1471-2148-11-80)
Supplement: Additional file 3 — Distribution of urea amidolyase, urea carboxylase, and amidase proteins in 64 fungal species. [file 1471-2148-11-80-S3.PDF]

Table S3. Distribution of urea amidolyase, urea carboxylase, and amidase proteins in 64 fungal species.[a]

Taxonomical group[b]	Species	Enzymes[c] UA	UC	A[d]
**[Zygomycota]**				
Zygomycetes/Mucorales	*Rhizopus oryzae* RA 99-880*	-	-	-
	Phycomyces blakesleeanus NRRL1555 v2.0	-	-	-
	Mucor circinelloides CBS277.49, v2.0	-	-	-
**[Chytridiomycota]**				
Chitridiomycetes/Chytridiales	*Batrachochytrium dendrobatidis* JEL423	-	-	-
**[Basidiomycota/Agaricomycotina]**				
Tremellomycetes /Tremellales	*Cryptococcus neoformans* H99*	-	1	-
Homobasidiomycetes/Agaricales	*Coprinus cinereus* okayama7#130*	-	-	-
	Laccaria bicolor S238N-H82	-	-	-
Homobasidiomycetes/Boletales	*Serpula lacrymans* S7.3 v2.0	-	1	-
**[Basidiomycota/Ustilaginomycotina]**				
Ustilaginomycetes/Ustilaginales	*Ustilago maydis* 521*	-	-	-
**[Basidiomycota/Pucciniomycotina]**				
Microbotryomycetes/Sporidiobolales	*Sporobolomyces roseus* v1.0	-	1	-
**[Ascomycota/Taphrinomycotina]**				
Schizosaccharomycetes/Schizosaccharomycetales	*Schizosaccharomyces pombe* 972h-*	-	-	-
**[Ascomycota/Pezizomycotina]**				
Eurotiomycetes/Onygenales	*Microsporum gypseum* CBS118893	-	-	-
	Microsporum canis CBS113480	-	-	-
	Trichophyton equinum CBS127.97	-	-	-
	Coccidioides immitis RS*	-	-	-
	Coccidioides immitis RMSCC 2394	-	-	-
	Coccidioides immitis RMSCC 3703	-	-	-
	Coccidioides immitis H538.4	-	-	-
	Coccidioides posadasii RMSCC 3488	-	-	-
	Coccidioides posadasii str. Silveira	-	-	-
	Histoplasma capsulatum G186AR	-	-	-
	Histoplasma capsulatum H143	-	-	-
	Histoplasma capsulatum H88	-	-	-
	Histoplasma capsulatum NAm1	-	-	-
	Blastomyces dermatitidis SLH14081	-	-	-
	Blastomyces dermatitidis ER-3	-	-	-
	Paracoccidioides brasiliensis Pb01	-	1	-
	Paracoccidioides brasiliensis Pb03	-	1	-
	Paracoccidioides brasiliensis Pb18	-	1	-
Eurotiomycetes/Eurotiales	*Aspergillus nidulans* FGSC A4*	-	1	-
	Aspergillus fumigatus Af293*	-	1	-
	Neosartorya fischeri NRRL 181	-	1	-

	Aspergillus terreus NIH2624*	-	1	-
	Aspergillus oryzae RIB40 / ATCC 42149*	-	-	-
	Aspergillus carbonarius ITEM 5010 v3	-	1	-
	Aspergillus clavatus NRRL 1	-	1	-
	Aspergillus flavus NRRL 3357	-	1	-
	Aspergillus niger ATCC 1015	-	1	1
Dothideomycetes/Capnodiales	*Mycosphaerella graminicola* v2.0*	-	-	1
	Mycosphaerella fijiensis v2.0	-	1	1
Dothideomycetes/Pleosporales	*Alternaria brassicicola* ATCC 96866	-	1	-
	Stagonospora nodorum SN15*	-	1	1
	Cochliobolus heterostrophus C5*	-	1	1
	Pyrenophora tritici-repentis Pt-1C-BFP	-	1	1
Leotiomycetes/Helotiales	*Botrytis cinerea* B05.10*	-	-	-
	Sclerotinia sclerotiorum 1980	-	-	1
Sordariomycetes/Sordariales	*Neurospora crassa* OR74A*	-	-	-
	Chaetomium globosum CBS 148.51	-	-	-
Sordariomycetes/Magnaporthales	*Magnaporthe oryzae* ATCC 64411*	1	-	(1)
Sordariomycetes/Hypocreales	*Nectria haematococca* v2.0*	1	1	(1)
	Fusarium graminearum PH-1 (NRRL 31084)*	1	-	(1)
	Fusarium oxysporum 4286*	1	1	(1)
	Fusarium verticillioides 7600*	1	1	(1)
	Trichoderma virens Gv29-8 v2.0	1	1	(1), 1
**[Ascomycota/Saccharomycotina]**				
Saccharomycetes/Saccharomycetales	*Yarrowia lipolytica* CLIB122*	2	-	(2)
	Candida albicans SC5314*	1	-	(1)
	Candida albicans WO1	1	-	(1)
	Candida parapsilosis isolate 317 from CDC	1	-	(1)
	Candida lusitaniae ATCC 42720*	1	-	(1)
	Debaryomyces hansenii CBS767*	1	-	(1)
	Ashbya gossypii ATCC 10895*	1	-	(1)
	Candida glabrata CBS138*	1	-	(1)
	Saccharomyces cerevisiae S288C*	1	-	(1)
	Saccharomyces cerevisiae RM11-1a	1	-	(1)

[a]See Additional file 4 (Table S4) for sequence sources.

[b]The phylum/subphylum (in square brackets) and class are given.

[c]See Figure 1 for the enzyme name abbreviations. The number of sequences found from each genome is shown. '-' indicates that no similar sequence was found.

[d]The amidase sequences that are a part of the urea amidolyase sequences are shown in parentheses.

*These fungal species are used in our further analysis.
