# Supplementary material for: Molecular evolution of urea amidolyase and urea carboxylase in fungi
Source: BMC Evol Biol. 2011 Mar 29;11:80. doi: 10.1186/1471-2148-11-80 (PMC3073912; doi:10.1186/1471-2148-11-80)
Supplement: Additional file 4 — Sequence sources of the urea amidolyase, urea carboxylase, and amidase from 64 fungal species. [file 1471-2148-11-80-S4.PDF]

Table S4. Sequence sources of the urea amidolyase, urea carboxylase, and amidase from 64 fungal species.

Taxonomical group[a]	Species	Source[b]	Enzymes[c] UA	UC	A[d]
**[Zygomycota]**					
Zygomycetes/ Mucorales	*Rhizopus oryzae* RA 99-880*	FGI	-	-	-
	Phycomyces blakesleeanus NRRL1555 v2.0	JGI	-	-	-
	Mucor circinelloides CBS277.49, v2.0	JGI	-	-	-
**[Chytridiomycota]**					
Chitridiomycetes/ Chytridiales	*Batrachochytrium dendrobatidis* JEL423	FGI	-	-	-
**[Basidiomycota/ Agaricomycotina]**					
Tremellomycetes / Tremellales	*Cryptococcus neoformans* H99*	JGI	-	CNAG_07944	-
Homobasidiomycetes / Agaricales	*Coprinus cinereus* okayama7#130*	JGI	-	-	-
	Laccaria bicolor S238N-H82	JGI	-	-	-
Homobasidiomycetes / Boletales	*Serpula lacrymans* S7.3 v2.0	JGI	-	169686	-
**[Basidiomycota/ Ustilaginomycotina]**					
Ustilaginomycetes/ Ustilaginales	*Ustilago maydis* 521*	FGI	-	-	-
**[Basidiomycota/ Pucciniomycotina]**					
Microbotryomycetes/ Sporidiobolales	*Sporobolomyces roseus* v1.0	JGI	-	21475	-
**[Ascomycota/ Taphrinomycotina]**					
Schizosaccharomycetes/ Schizosaccharomycetales	*Schizosaccharomyces pombe* 972h-*	Sanger	-	-	-
**[Ascomycota/ Pezizomycotina]**					
Eurotiomycetes/ Onygenales	*Microsporum gypseum* CBS118893	FGI	-	-	-
	Microsporum canis CBS113480	FGI	-	-	-
	Trichophyton equinum CBS127.97	FGI	-	-	-
	Coccidioides immitis RS*	FGI	-	-	-
	Coccidioides immitis RMSCC 2394	FGI	-	-	-
	Coccidioides immitis RMSCC 3703	FGI	-	-	-
	Coccidioides immitis H538.4	FGI	-	-	-
	Coccidioides posadasii RMSCC 3488	FGI	-	-	-
	Coccidioides posadasii str. Silveira	FGI	-	-	-
	Histoplasma capsulatum G186AR	FGI	-	-	-
	Histoplasma capsulatum H143	FGI	-	-	-
	Histoplasma capsulatum H88	FGI	-	-	-
	Histoplasma capsulatum NAm1	FGI	-	-	-
	Blastomyces dermatitidis SLH14081	FGI	-	-	-
	Blastomyces dermatitidis ER-3	FGI	-	-	-
	Paracoccidioides brasiliensis Pb01	FGI	-	PAAG_02163	-
	Paracoccidioides brasiliensis Pb03	FGI	-	PABG_02398	-

	Paracoccidioides brasiliensis Pb18	FGI	-	PADG_00734	-
Eurotiomycetes/ Eurotiales	*Aspergillus nidulans* FGSC A4*	FGI	-	ANID_00887T0	-
	Aspergillus fumigatus Af293*	FGI	-	Afu1g15520	-
	Neosartorya fischeri NRRL 181	FGI	-	NFIA_009890	-
	Aspergillus terreus NIH2624*	FGI	-	ATET_05246	-
	Aspergillus oryzae RIB40 / ATCC 42149*	FGI	-	-	-
	Aspergillus carbonarius ITEM 5010 v3	JGI	-	10485	-
	Aspergillus clavatus NRRL 1	FGI	-	ACLA_019830	-
	Aspergillus flavus NRRL 3357	FGI	-	AFL2T_01101	-
	Aspergillus niger ATCC 1015	FGI	-	e_gw1_1.1117	fge1_pg_C_12000388
Dothideomycetes/ Capnodiales	*Mycosphaerella graminicola* v2.0*	JGI	-	-	75341
	Mycosphaerella fijiensis v2.0	JGI	-	41182	82172
Dothideomycetes/ Pleosporales	*Alternaria brassicicola* ATCC 96866	JGI	-	AB06360.1	-
	Stagonospora nodorum SN15*	FGI	-	SNOT_02186	SNOT_08324
	Cochliobolus heterostrophus C5*	JGI	-	57707	29777
	Pyrenophora tritici-repentis Pt-1C-BFP	JGI	-	PTRG_09405	PTRG_11638
Leotiomycetes/ Helotiales	*Botrytis cinerea* B05.10*	FGI	-	-	-
	Sclerotinia sclerotiorum 1980	FGI	-	-	SS1T_04628
Sordariomycetes/ Sordariales	*Neurospora crassa* OR74A*	FGI	-	-	-
	Chaetomium globosum CBS 148.51	FGI	-	-	-
Sordariomycetes/ Magnaporthales	*Magnaporthe oryzae* ATCC 64411*	FGI	MGG_04386	-	
Sordariomycetes/ Hypocreales	*Nectria haematococca* v2.0*	JGI	79968	44732	
	Fusarium graminearum PH-1 (NRRL 31084)*	FGI	FGSG_10913	-	
	Fusarium oxysporum 4286*	FGI	FOXG_12848	FOXG_07646	
	Fusarium verticillioides 7600*	FGI	FVEG_11593T0	FVEG_04571T0	
	Trichoderma virens Gv29-8 v2.0	JGI	53233	67729	42211
**[Ascomycota/ Saccharomycotina]**					
Saccharomycetes/ Saccharomycetales	*Yarrowia lipolytica* CLIB122*	Géno	YALI0E07271g YALI0E35156g	-	
	Candida albicans SC5314*	CGD	orf19_780	-	
	Candida albicans WO1	FGI	CAWT_00928	-	
	Candida parapsilosis isolate 317 from CDC	FGI	CPAG_03627	-	
	Candida lusitaniae ATCC 42720*	FGI	CLUT_00442	-	
	Debaryomyces hansenii CBS767*	Géno	DEHA2D07040g	-	
	Ashbya gossypii ATCC 10895*	NCBI	45187924	-	
	Candida glabrata CBS138*	Géno	CAGL0M05533g	-	
	Saccharomyces cerevisiae S288C*	SGD	YBR208C	-	
	Saccharomyces cerevisiae RM11-1a	FGI	SCRT_02761	-	

[a]The phylum/subphylum (in square brackets) and class/order are given.

[b]FGI: Fungal Genome Initiative (http://www.broadinstitute.org/science/projects/fungal-genome-initiative/fungal-genome-initiative), JGI: Joint Genome Institute (http://www.jgi.doe.gov), Sanger: The *S. pombe* Genome Project (http://www.sanger.ac.uk/Projects/Fungi/), Géno: Génolevures Genomic Exploration of the Hemiascomycete Yeasts (http://www.genolevures.org]), CGD: Candida Genome Database (http://www.candidagenome.org/), NCBI: National Center for Biotechnology Information (http://www.ncbi.nlm.nih.gov), and SGD: Saccharomyces Genome Database (http://www.yeastgenome.org/).

[b]See Figure 1 for the enzyme name abbreviations. '-' indicates that no similar sequence was found.

*These fungal species are used in our further analysis.
