# Supplementary material for: Molecular evolution of urea amidolyase and urea carboxylase in fungi
Source: BMC Evol Biol. 2011 Mar 29;11:80. doi: 10.1186/1471-2148-11-80 (PMC3073912; doi:10.1186/1471-2148-11-80)
Supplement: Additional file 5 — Sequence sources of the urease, methylcrotonoyl-CoA carboxylase, and propionyl-CoA carboxylase from the selected 27 fungal species. [file 1471-2148-11-80-S5.PDF]

Table S5. Sequence sources of the urease, methylcrotonoyl-CoA carboxylase, and propionyl-CoA carboxylase from the selected 27 fungal species.[a]

	Enzymes[b]		
**Species**	**Urease**	**MccA**	**PccA**
Rhizopus oryzae RA 99-880	RO3G_06489	RO3G_06576	RO3G_06560
Ustilago maydis 521	UM06045	UM04382	-
Cryptococcus neoformans H99	CNAG_05540	CNAG_01680	-
Coprinus cinereus okayama7#130	CC1G_10059	CC1G_13741	CC1G_05511
Schizosaccharomyces pombe 972h-	SPAC1952_11c	-	-
Coccidioides immitis RS	CIMT_05193	CIMT_07030	CIMT_05331
Aspergillus nidulans FGSC A4	AN10079	AN4690	AN7764
Aspergillus fumigatus Af293	Afu1g04560	Afu5g08910	Afu5g07580
Aspergillus terreus NIH2624	ATET_03748	ATET_06576	ATET_08368
Aspergillus oryzae RIB40 / ATCC 42149	AO090003000879	AO090020000495	-
Mycosphaerella graminicola v2.0	85598	70525	109805
Stagonospora nodorum SN15	SNOT_11285	SNOT_09555	SNOT_12342
Cochliobolus heterostrophus C5	95543	78650	105664
Botritis cinerea B05.10	BC1T_13063	BC1T_08870	BC1T_02620
Neurospora crassa OR74A	NCU03127	NCU00591	-
Magnaporthe oryzae ATCC 64411	MGG_01324	MGG_10320	-
Nectria haematococca v2.0	65875	92030	-
Fusarium graminearum PH-1 (NRRL 31084)	FGSG_00740<br>FGSG_10627	FGSG_08688	-
Fusarium oxysporum 4286	FOXG_01071<br>FOXG_17146	FOXG_03110	-
Fusarium verticillioides 7600	FVEG_00443	FVEG_01973	-
Yarrowia lipolytica CLIB122	-	YALI0B14619g	-
Candida albicans SC5314	-	-	-
Candida lusitaniae ATCC 42720	-	-	-
Debaryomyces hansenii CBS767	-	-	-
Ashbya gossypii ATCC 10895	-	-	-
Candida glabrata CBS138	-	-	-
Saccharomyces cerevisiae S288C	-	-	-

[a]See Additional file 4 (Table S4) for the data source for each genome.

[b]See Figure 1 for the enzyme name abbreviations. The sequences IDs found from each genome is shown. '-' indicates that no similar sequence was found.
