# Supplementary material for: Molecular evolution of urea amidolyase and urea carboxylase in fungi
Source: BMC Evol Biol. 2011 Mar 29;11:80. doi: 10.1186/1471-2148-11-80 (PMC3073912; doi:10.1186/1471-2148-11-80)
Supplement: Additional file 6 — Maximum-likelihood phylogeny of the carboxylation-domain sequences from urea carboxylase, urea amidolyase, methylcrotonoyl-CoA carboxylase, propionyl-CoA carboxylase, pyruvate carboxylase, and acetyl-CoA carboxylase. [file 1471-2148-11-80-S6.PDF]

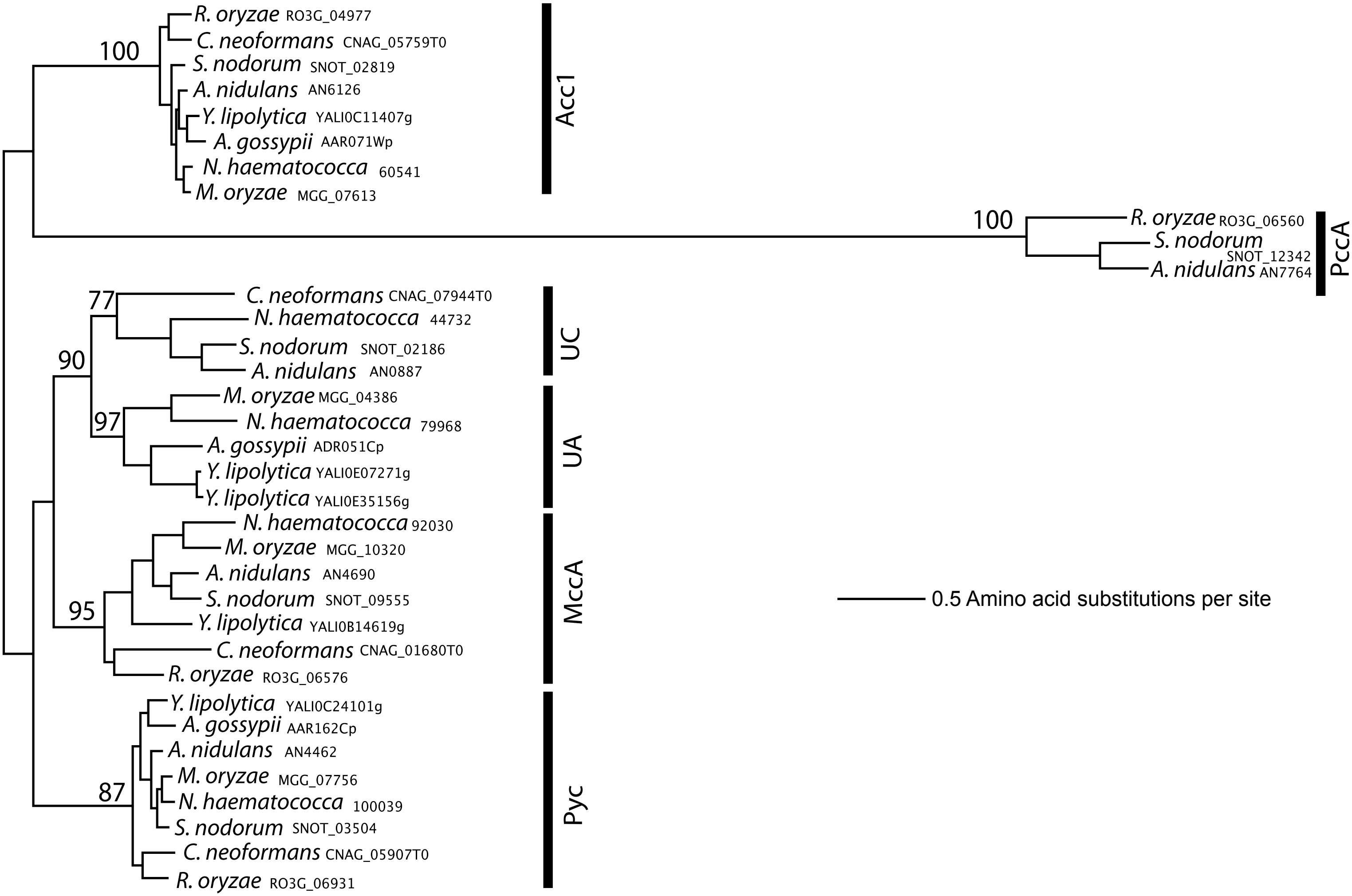

Figure S1. Maximum-likelihood phylogeny of carboxylation domain sequences. The maximum-likelihood phylogeny was reconstructed using the carboxylation domain sequences of the following fungal proteins : urea amidolyase (UA), urea carboxylase (UC), acetyl-CoA carboxylase (Acc1), propionyl-CoA carboxylase (PccA), methylcrotonoyl-CoA carboxylase (MccA) and pyruvate carboxylase (PyC). The numbers above the internal branches show bootstrap values (%). Only values ≥ 70 are shown.
