# Supplementary material for: Molecular evolution of urea amidolyase and urea carboxylase in fungi
Source: BMC Evol Biol. 2011 Mar 29;11:80. doi: 10.1186/1471-2148-11-80 (PMC3073912; doi:10.1186/1471-2148-11-80)
Supplement: Additional file 7 — Sequence sources of urea amidolyase, urea carboxylase, and amidase in eubacterial genomes. [file 1471-2148-11-80-S7.PDF]

Table S6. Sequence sources of urea amidolyase, urea carboxylase, and amidase in eubacterial genomes.

Phylum or Class	Species	ACC#[a]	Enzymes[b]			
			UA	UC	A	Urease
Alphaproteobacteria	*Caulobacter crescentus NA1000*	NC_011916	-	221234842	221234843	-
	Asticcacaulis excentricus CB 48	NZ_ACQR00000000	-	241771960	241771961	-
	Sinorhizobium medicae WSM419	NC_009636	-	-	-	150397583<br>150397586<br>150397588
Betaproteobacteria	*Achromobacter piechaudii ATCC 43553*	NZ_ADMS00000000	-	293607215	293607216	-
	Bordetella pertussis Tohama I	NC_002929	-	-	-	33594086<br>33594087<br>33594089
	Nitrosomonas europaea ATCC 19718	NC_004757	-	30250344<br>30250340*	-	-
	Neisseria meningitidis FAM18	NC_008767	-	-	-	-
	Burkholderia sp. CCGE1001	NZ_ADDJ00000000	-	282888296	282888297	282888448<br>282888449<br>282888450
Gammaproteobacteria	*Escherichia coli O111:H- str. 11128*	NC_013364	-	-	-	260867324<br>260867323<br>260867322
	Yersinia pestis Angola	NC_010159	-	-	-	162421917<br>162421306
	Haemophilus influenzae 86-028NP	NC_007146	-	-	-	68249136<br>68249137<br>68249138
	Pantoea ananatis LMG 20103	NC_013956	291616199	291619625	291616199	-
	Pantoea sp. At-9b	NZ_ACYJ00000000	-	258639802<br>258639881	258639803	-
	Shewanella oneidensis MR-1	NC_004347	-	-	-	-
	Pseudomonas aeruginosa LESB58	NC_011770	-	-	-	218893963<br>218893960<br>218893962
	Coxiella burnetii Dugway 5J108-111	NC_009727	-	-	-	-
	Pectobacterium carotovorum subsp. carotovorum PC1	NC_012917	-	253688548<br>253688770	253688549	-
	Xanthomonas campestris pv. campestris str. B100	NC_010688	-	-	-	-

	Cellvibrio japonicus Ueda107	NC_010995	-	192360305<br>192360281	192360851	-
	Teredinibacter turnerae T7901	NC_012997	-	254787389	254787390	254788040<br>254788041<br>254788042
	Marinomonas sp. MED121	NZ_AANE00000000	-	87119094	87119095	87120670<br>87120669
	Klebsiella pneumoniae 342	NC_011283	-	206578981	206581101	206580665<br>206579658<br>206580264
	Pseudomonas fluorescens SBW25	NC_012660	-	-	-	229588129<br>229588133<br>229588130
Deltaproteobacteria	*Geobacter sp. M21*	NC_012918	-	-	-	-
	Sorangium cellulosum 'So ce 56'	NC_010162	-	162453191	-	162454831<br>162454830
Epsilonproteobacteria	*Helicobacter pylori B38*	NC_012973	-	-	-	254778798<br>254778799
	Wolinella succinogenes DSM 1740	NC_005090	-	34557492	34557494	-
Acidobacteria	*Acidobacterium capsulatum ATCC 51196*	NC_012483	-	-	-	-
	Solibacter usitatus Ellin6076	NC_008536	-	116619994	116619993	-
Cyanobacteria	*Synechococcus sp. PCC 7002*	NC_010475	-	-	-	170076824<br>170079032<br>170077934
	Gloeobacter violaceus PCC 7421	NC_005125	-	37520527	37520530	-
	Cyanothece sp. PCC 7425	NC_011884	-	220907713	220908629	220907679<br>220907680<br>220907681
Deinococcus-Thermus	*Thermus thermophilus HB8*	NC_006461	-	-	-	-
	Deinococcus deserti VCD115	NC_012526	-	-	-	-
Chloroflexi	*Dehalococcoides ethenogenes 195*	NC_002936	-	-	-	-
Aquificae	*Aquifex aeolicus VF5*	NC_000918	-	-	-	-
Thermotogae	*Thermotoga maritima MSB8*	NC_000853	-	-	-	-
Fusobacteria	*Fusobacterium nucleatum subsp. nucleatum ATCC 25586*	NC_003454	-	-	-	-
Verrucomicrobia	*Verrucomicrobium spinosum DSM 4136*	NZ_ABIZ00000000	-	171912641	171912640	171911815<br>171911816<br>171911817
Chlamydiae	*Chlamydophila pneumoniae CWL029*	NC_000922	-	-	-	-

	Chlamydia trachomatis B/TZ1A828/OT	NC_012687	-	-	-	-
Bacterioidetes	*Porphyromonas gingivalis W83*	NC_002950	-	-	-	-
Chlorobi	*Chlorobium limicola DSM 245*	NC_010803	-	-	-	-
Fibrobacteres	*Fibrobacter succinogenes subsp. succinogenes S85*	NC_013410	-	-	-	-
Actinobacteria	*Mycobacterium tuberculosis F11*	NC_009565	-	-	-	148823061<br>148823060<br>148823059
	Corynebacterium aurimucosum ATCC 700975	NC_012590	-	-	-	-
	Streptomyces avermitilis MA-4680	NC_003155	-	29833240	29833239	29833648<br>29829257<br>29829258<br>29833647<br>29833646
	Bifidobacterium longum subsp. infantis ATCC 15697	NC_011593	-	-	-	213691032<br>213691031
Spirochaetes	*Borrelia burgdorferi ZS7*	NC_011728	-	-	-	-
	Treponema denticola ATCC 35405	NC_002967	-	-	-	-
Planctomycetes	*Rhodopirellula baltica SH 1*	NC_005027	-	-	-	-
Firmicutes	*Clostridium botulinum A2 str. Kyoto*	NC_012563	-	-	-	-
	Mycoplasma hyopneumoniae 7448	NC_007332	-	-	-	-
	Streptococcus pneumoniae 70585	NC_012468	-	-	-	-
	Bacillus anthracis str. CDC 684	NC_012581	-	-	-	-
	Roseburia intestinalis L1-82	NZ_ABYJ00000000	-	240144639	240144640	-

[a]All the bacterial sequences were downloaded from National Center for Biotechnology Information (http://www.ncbi.nlm.nih.gov/).

[b]See Figure 1 for the enzyme name abbreviations. '-' indicates that no similar sequence was found.

*This UC sequence was only 780 amino acids long, consisting of incomplete urea carboylase domain. Hence it was not used in phylogenetic analysis.
