# Supplementary material for: Molecular evolution of urea amidolyase and urea carboxylase in fungi
Source: BMC Evol Biol. 2011 Mar 29;11:80. doi: 10.1186/1471-2148-11-80 (PMC3073912; doi:10.1186/1471-2148-11-80)
Supplement: Additional file 8 — Distance between amidase and urea carboxylase genes in eubacterial genomes. [file 1471-2148-11-80-S8.PDF]

Table S7. Distance between amidase and urea carboxylase genes in eubacterial genomes.

Species	No. of bp between UC and A[a]
Caulobacter crescentus NA1000	-2 (0)
Asticcacaulis excentricus CB 48	0 (0)
Achromobacter piechaudii ATCC 43553[b]	18 (0)
Burkholderia sp. CCGE1001	61 (0)
Pantoea sp. At-9b[b]	2 (0)
Pectobacterium carotovorum subsp. carotovorum PC1[b]	-6 (0)
Cellvibrio japonicus Ueda107	118 (0)
Teredinibacter turnerae T7901	-6 (0)
Marinomonas sp. MED121	25 (0)
Klebsiella pneumoniae 342	-2 (0)
Wolinella succinogenes DSM 1740	943 (1)
Solibacter usitatus Ellin6076	2 (0)
Gloeobacter violaceus PCC 7421	1,701 (2)
Cyanothece sp. PCC 7425	979,743 (916)
Verrucomicrobium spinosum DSM 4136	118 (0)
Streptomyces avermitilis MA-4680	16 (0)
Roseburia intestinalis L1-82	16 (0)

[a]See Figure 1 for the enzyme name abbreviations. The number of genes present between UC and A genes are given in parentheses. Negative distances indicate that these two genes are overlapped.

[b]These species have two copies of the urea carboxylase (UC) gene. The UC gene in this table is the one that is closest to the A gene in the respective genome.
