# Supplementary material for: Molecular evolution of urea amidolyase and urea carboxylase in fungi
Source: BMC Evol Biol. 2011 Mar 29;11:80. doi: 10.1186/1471-2148-11-80 (PMC3073912; doi:10.1186/1471-2148-11-80)
Supplement: Additional file 9 — Minimum-evolution phylogeny of amidase protein sequences. [file 1471-2148-11-80-S9.PDF]

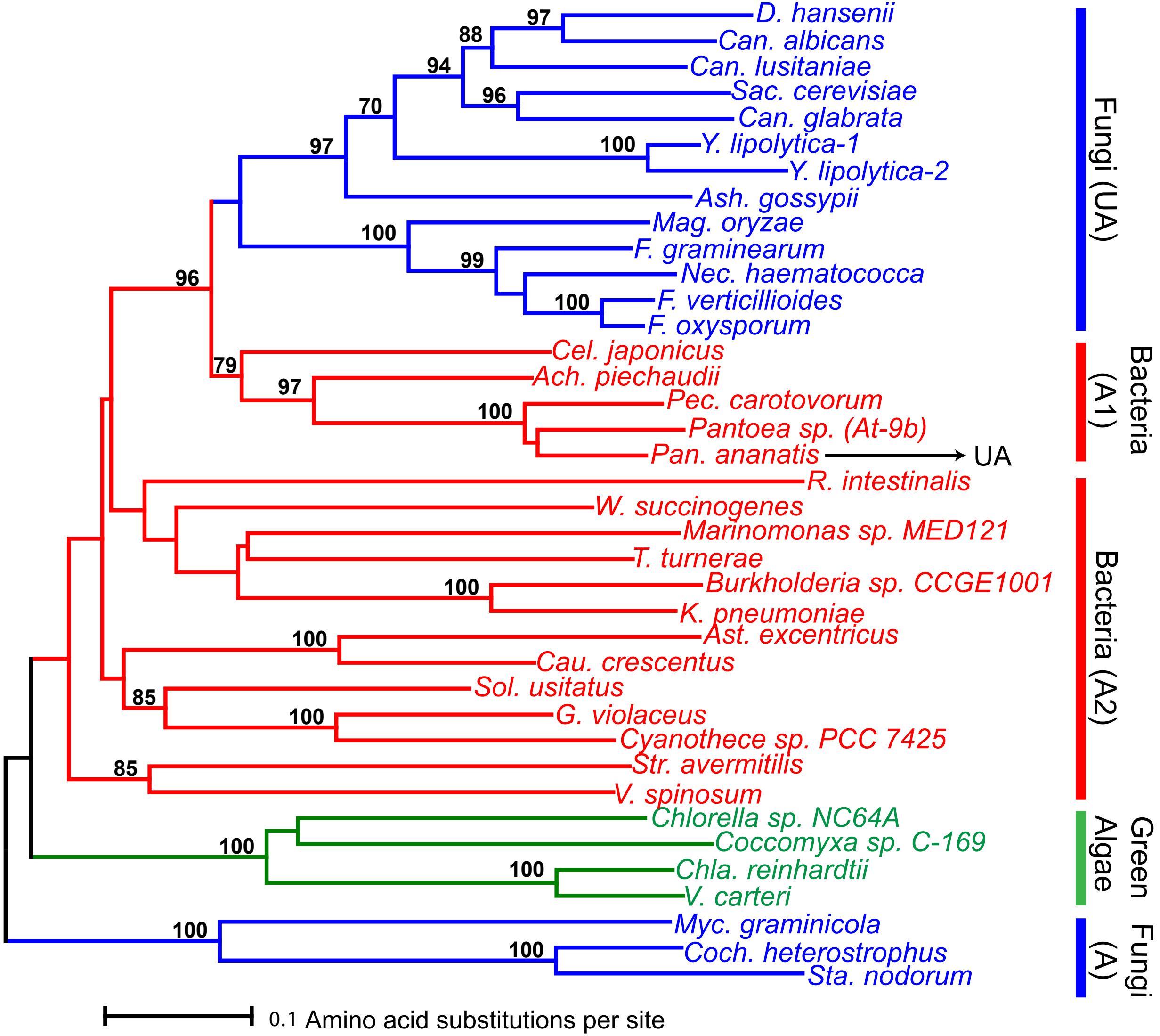

Figure S2. Minimum-evolution phylogeny of amidase protein sequences. The minimum-evolution phylogeny was reconstructed using the protein sequences from the amidase domains of the urea amidolyase proteins and the amidase proteins. The numbers above the internal branches show bootstrap values (%). Only values ≥ 70 are shown. Branches are colored as follows: blue for fungi, green for green algae, and red for bacteria. The bacterial urea carboxylase groups denoted by A1 and A2 correspond with the same groups in Figure 3.
