# Supplementary material for: Molecular evolution of urea amidolyase and urea carboxylase in fungi
Source: BMC Evol Biol. 2011 Mar 29;11:80. doi: 10.1186/1471-2148-11-80 (PMC3073912; doi:10.1186/1471-2148-11-80)
Supplement: Additional file 10 — Minimum evolution phylogeny of urea carboxylase protein sequences. [file 1471-2148-11-80-S10.PDF]

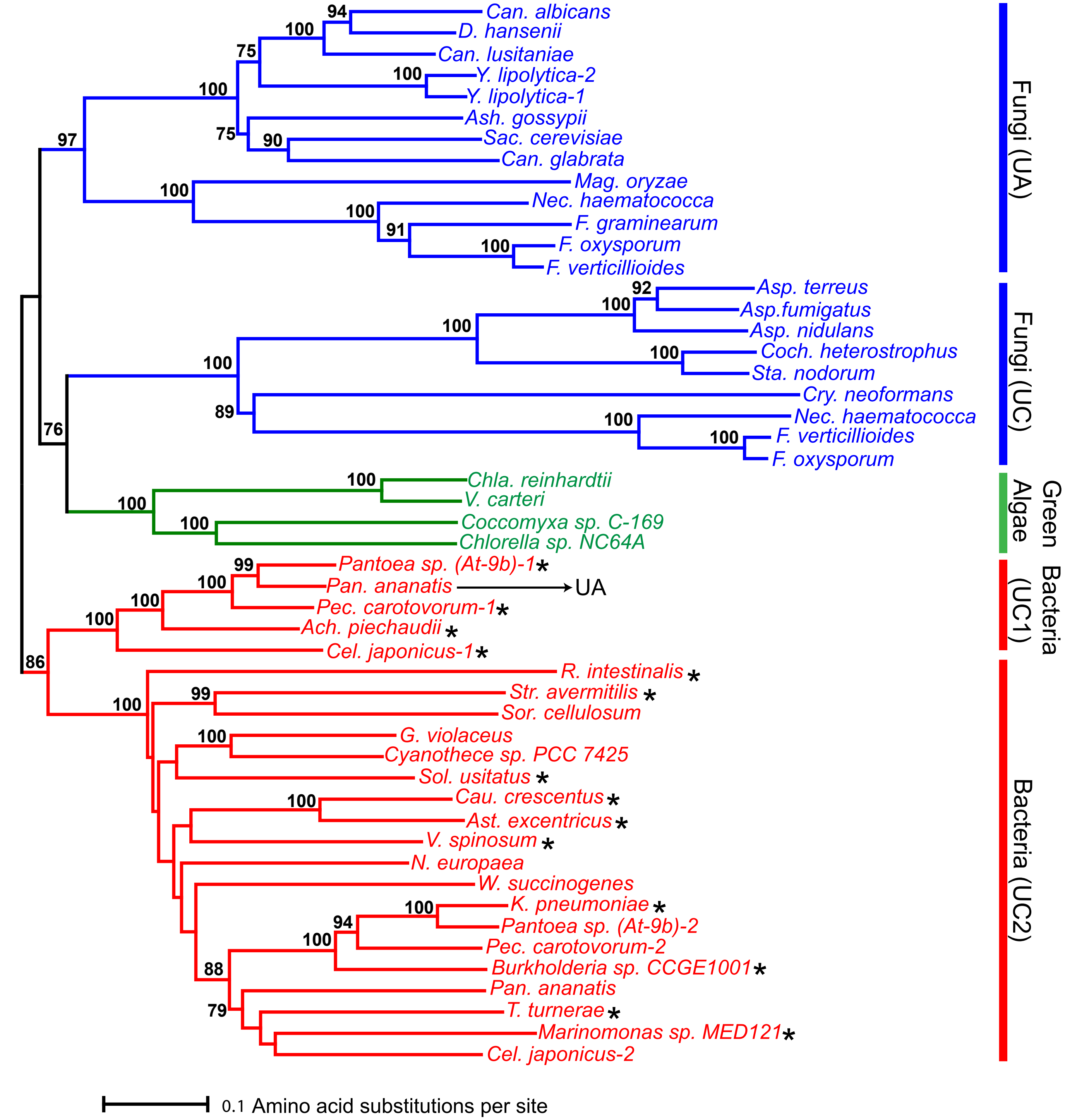

Figure S3. Minimum-evolution phylogeny of urea carboxylase protein sequences. The minimum-evolution phylogeny was reconstructed using the protein sequences from the urea-carboxylase domains of the urea amidolyase proteins and the urea carboxylase proteins. The numbers above the internal branches show bootstrap values (%). Only values ≥ 70 are shown. Branches are colored as follows: blue for fungi, green for green algae, and red for bacteria. The bacterial urea carboxylase groups denoted by UC1 and UC2 correspond with the same groups in Figure 4. The asterisks beside the bacterial names indicate that their urea carboxylase genes are next to the amidase genes in their genomes.
