# Supplementary material for: Molecular evolution of urea amidolyase and urea carboxylase in fungi
Source: BMC Evol Biol. 2011 Mar 29;11:80. doi: 10.1186/1471-2148-11-80 (PMC3073912; doi:10.1186/1471-2148-11-80)
Supplement: Additional file 11 — Maximum-likelihood phylogeny of urea carboxylase protein sequences including the two Hydra sequences. [file 1471-2148-11-80-S11.PDF]

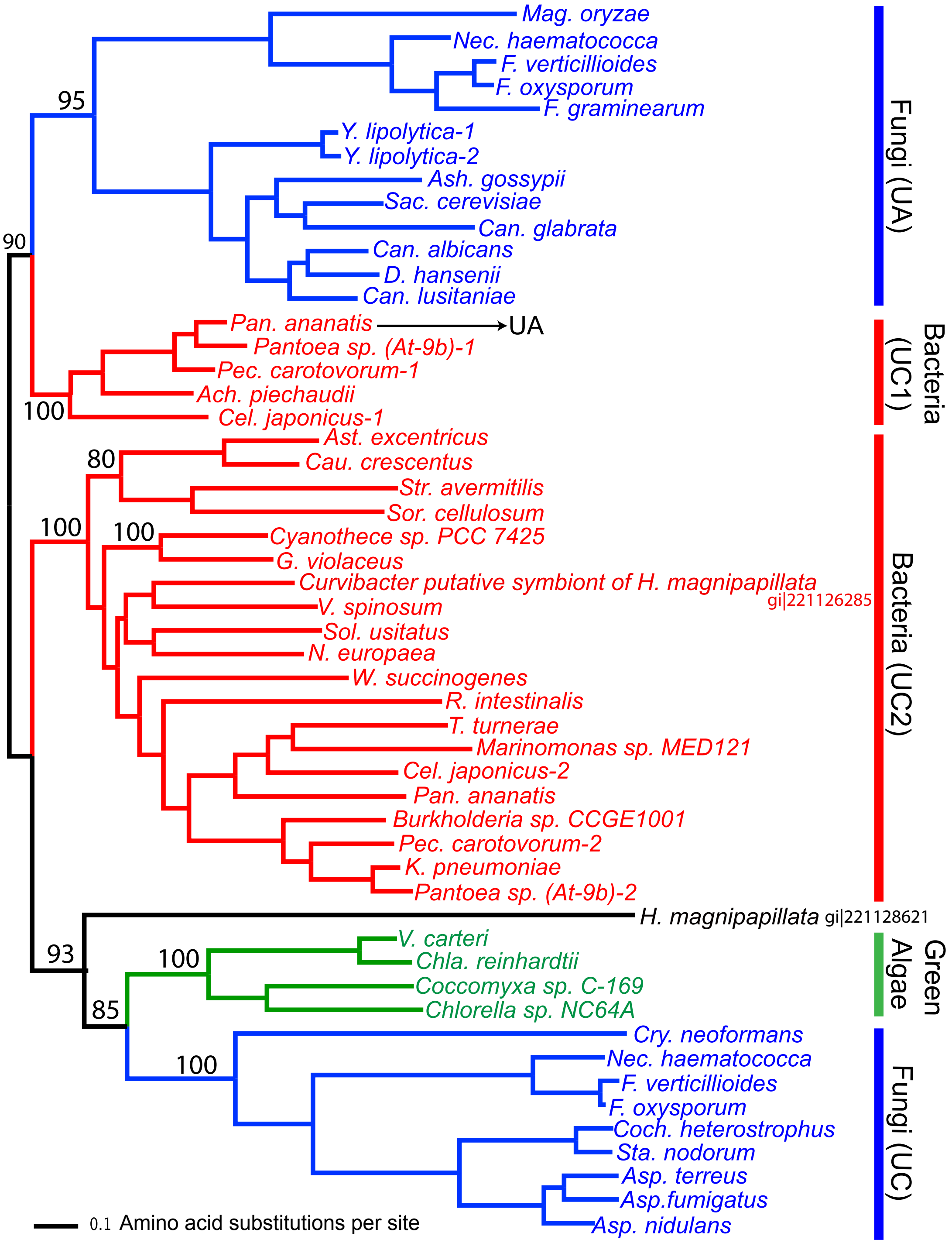

Figure S4. Maximum-likelihood phylogeny of urea carboxylase protein sequences including the two sequences found in *Hydra magnipapillata*. The maximum-likelihood phylogeny was reconstructed using the protein sequences from the urea carboxylase domains of the urea amidolyase proteins and the urea carboxylase proteins. The numbers above or below the internal branches show bootstrap values (%). Only bootsrap values equal to or higher than 70% are shown. Branches are colored as follows: black for metazoan, blue for fungi, green for green algae, and red for bacteria.
